# Liver Organoids: Recent Developments, Limitations and Potential

**DOI:** 10.3389/fmed.2021.574047

**Published:** 2021-05-05

**Authors:** Sean Philip Harrison, Saphira Felicitas Baumgarten, Rajneesh Verma, Oleg Lunov, Alexandr Dejneka, Gareth John Sullivan

**Affiliations:** ^1^Hybrid Technology Hub–Center of Excellence, Institute of Basic Medical Sciences, University of Oslo, Oslo, Norway; ^2^Department of Pediatric Research, Oslo University Hospital, Oslo, Norway; ^3^Institute of Physics of the Czech Academy of Sciences, Prague, Czechia; ^4^Norwegian Center for Stem Cell Research, Oslo University Hospital, University of Oslo, Oslo, Norway; ^5^Institute of Immunology, Oslo University Hospital, Oslo, Norway

**Keywords:** pluripotent stem cells, stem cell differentiation, liver development, liver architecture, 3D microscopy, organoids

## Abstract

Liver cell types derived from induced pluripotent stem cells (iPSCs) share the potential to investigate development, toxicity, as well as genetic and infectious disease in ways currently limited by the availability of primary tissue. With the added advantage of patient specificity, which can play a role in all of these areas. Many iPSC differentiation protocols focus on 3 dimensional (3D) or organotypic differentiation, as these offer the advantage of more closely mimicking *in vivo* systems including; the formation of tissue like architecture and interactions/crosstalk between different cell types. Ultimately such models have the potential to be used clinically and either with or more aptly, in place of animal models. Along with the development of organotypic and micro-tissue models, there will be a need to co-develop imaging technologies to enable their visualization. A variety of liver models termed “organoids” have been reported in the literature ranging from simple spheres or cysts of a single cell type, usually hepatocytes, to those containing multiple cell types combined during the differentiation process such as hepatic stellate cells, endothelial cells, and mesenchymal cells, often leading to an improved hepatic phenotype. These allow specific functions or readouts to be examined such as drug metabolism, protein secretion or an improved phenotype, but because of their relative simplicity they lack the flexibility and general applicability of *ex vivo* tissue culture. In the liver field these are more often constructed rather than developed together organotypically as seen in other organoid models such as brain, kidney, lung and intestine. Having access to organotypic liver like surrogates containing multiple cell types with *in vivo* like interactions/architecture, would provide vastly improved models for disease, toxicity and drug development, combining disciplines such as microfluidic chip technology with organoids and ultimately paving the way to new therapies.

## Introduction

Organoids are *in vitro* cellular systems that self-organize through mechanisms similar to *in vivo*, they recapitulate the structure and in many cases the function of the *in vivo* tissue in question, ultimately providing utility in both the clinical and basic research arenas (1, 2). An ever-expanding number of examples of organoid models that have been derived from both adult primary cells and pluripotent stem cells (PSCs) (3, 4). In this review we will focus on the recent developments in liver based organoid models from iPSCs, highlighting both potential and limitations, with a brief comparisons to other areas of the organoid field. An advantage of deriving organoids from primary material is that the starting material can be sourced with the desired phenotype and functional maturity, this being a limitations of PSC derived progeny which tend to have mixed fetal/adult features (5, 6). However, access to primary tissue still remains a fundamental barrier, especially with respect to organs where invasive surgery is required for sampling. Thus, giving credence to the utilization of PSCs, as the attributes of both pluripotency and self-renewal provide a solution to the dearth of primary material albeit with the above limitations.

Some examples of the potential myriad uses for discrete *in vitro* tissue units include model systems for both disease and development. In contrast to current monolayer models a successfully differentiated organoid can mimic the multicellular complexity and 3D architecture that provides normal organ function including cell to cell and cell to ECM interactions (7–9). Both toxicity and drug development are areas requiring improved hepatic models. The liver is the primary site of xenobiotic metabolism and hepatotoxicity is a leading cause of drug development failure (10). Here organoids can offer a potential advantage over non-human models as they can examine species-specific effects with the aim of reducing or replacing the animal models currently used, importantly addressing potential species differences. In fact the U.S. Environmental Protection Agency announced plans to end the use of dogs, mice, rabbits, and other mammals for chemical and pesticide testing by 2035 (https://www.epa.gov/research/administrator-memo-prioritizing-efforts-reduce-animal-testing-september-10-2019). This carries with it obvious ethical advantages related to animal welfare as well as offering better translation to clinical trials and removing key physiological species differences in terms of expression of drug metabolizing enzymes such as the cytochrome P450s (CYP450s) (11). Here too is an area where the use of iPSC technology could find a particular niche. For example, the genes encoding the CYP450 enzymes are sites of genetic variability within human populations leading to a spectrum of phenotypes from ultrafast-metabolizers to poor metabolism of certain drugs and compounds (12). For example CYP450 2D6 converts codeine to morphine, pain relief may be minimal for a poor metabolizer while an ultra-rapid metabolizers administered a “normal dose” could lead to a life threatening overdose of morphine (13). Differentiating organoids from iPSCs derived from these different populations would potentially allow for a more “personalized” use of current drug regimes and the ability to better target future drug development. Extending this further, iPSC derived organoids could offer highly personalized patient specific drug testing. Similarly these techniques can be used to model genetic disease in an organ specific manner using iPSCs derived from donors of a certain genotype to generate the required organoids or by introducing the genetic lesions into control iPSCs, using genome editing approaches such as clustered regularly interspaced short palindromic repeats (CRISPR) (14, 15).

Further developments could allow organoids to play a role in regenerative medicine with the ultimate goal of providing transplantable organs or be used to seed bio-artificial liver devices, analogous to kidney dialysis machines, or form the active functional unit of an implantable device, thereby providing temporary function allowing the livers highly regenerative capacity to repair itself by relieving the burden (16). Current organoid systems tend to be carried out at the lab scale, which is not in alignment with the above requirement of massive amounts of organoids. Some groups have addressed this issue and have moved to scaling the process (17). This goal however is severely hampered by being prohibitively expensive to produce the required numbers of organoids, due to the heavy reliance of recombinant growth factors. Further considerations in this area include the need to develop human sized individual units of tissue, as demonstrated with heart sheets (18), and the associated requirement for oxygen, nutrients, and vascularization (either artificial or cell based) these larger units would necessitate, as current protocols tend to generate organoids at the micrometer scale. Tissue engineering techniques can be envisioned using organoids as the fundamental building blocks to construct bigger organs, combined with the use of ECM and/or biomaterials as scaffolds. Another area gaining momentum is “Organ-on-Chip” technology, combining organoid technology with the control and automation possible with chip-based technology (19, 20). Also providing a platform to both characterize and analyse the organoids function in an “online” format, allowing individual tissue types (organoids) to be grown in the same systems facilitating the construction of more complex models.

The following review will provide an overview of liver development and architecture and how current organoid models have been developed, with *in vivo* development in mind. In addition, why there is a necessity for these models, highlighting current model limitations, also we will cover current application of organotypic approaches in disease modeling. Finally we will address the status of optical imaging approaches to visualize large 3D structures. However, we will primarily focus on liver organoid models and will not describe the other tissue models that are available, but one can explore some examples of different organoid models in the following reviews (3, 21–24).

## Main

### Liver Development and Architecture

*In vitro* protocols for differentiating PSCs to hepatic cells rely on prior knowledge of how a liver develops during embryogenesis. Beginning with the formation of the three germ layers which occurs during gastrulation, the definitive endoderm (DE) arises via an epithelial to mesenchymal transition (EMT) at the anterior of the primitive streak in response to WNT and Nodal signaling (25–27). The DE then folds to form the gut tube (28) of which, the ventral foregut is the site of hepatogenesis (29, 30). Hepatic specification is induced by signaling from two adjacent mesodermal sources the cardiac mesoderm and the *septum transversum mesenchyme* (STM) (31–33), leading to the formation of hepatoblasts, which are a bipotent cell type that give rise to the parenchymal cells of the liver, namely the hepatocytes and cholangiocytes (34, 35). On formation of the liver bud, it is colonized by hematopoietic cells that produce the cytokine, oncostatin M (OSM) which along with glucocorticoid hormones, HGF and Wnt, drive liver maturation (36), this process of maturation continues throughout the postnatal period (37).

**Figure 1:**
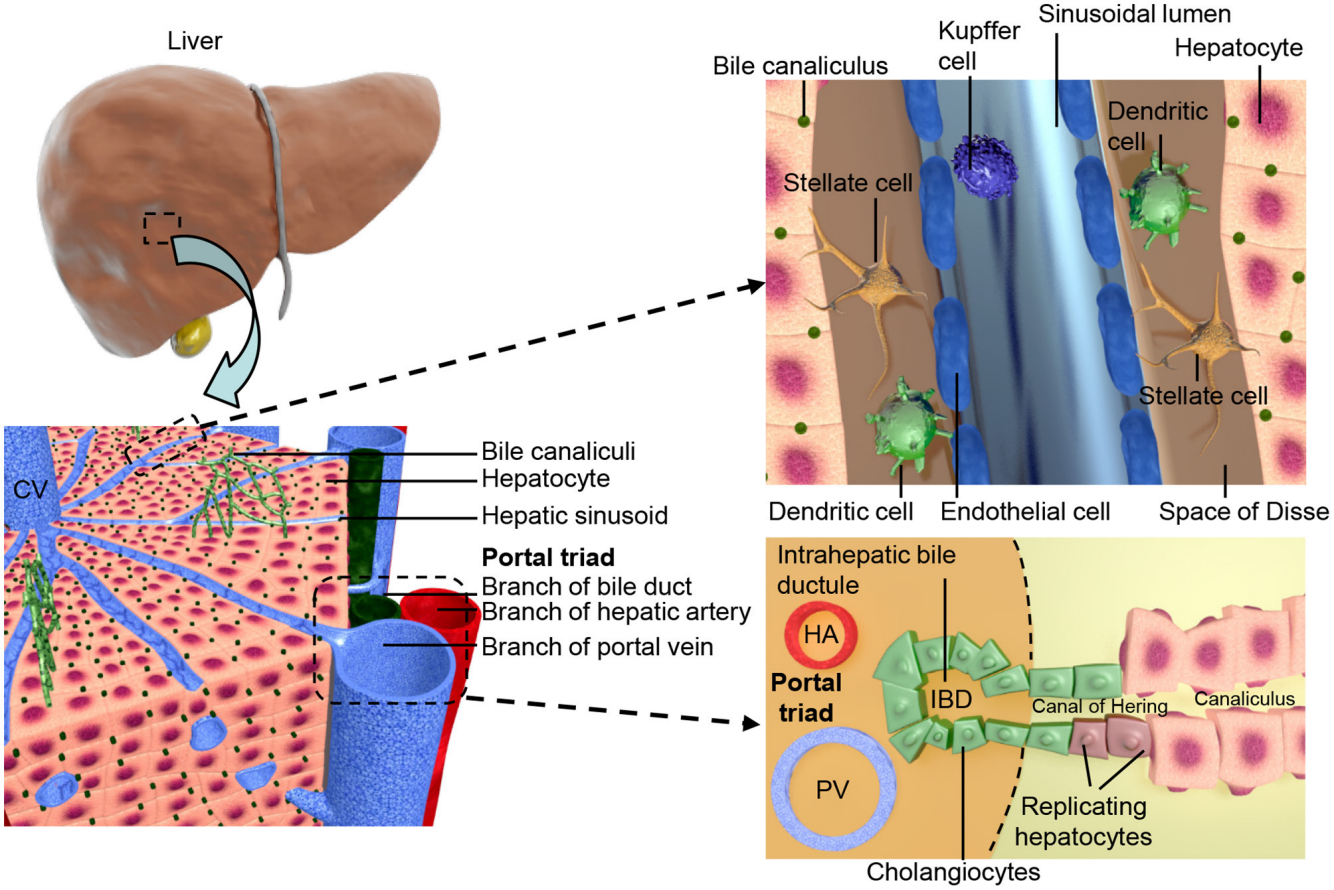
Shows the structural organization of the liver at different scales. The two lobes of the liver consist of hexagonal units known as lobules, these consist of a central vein surrounded by portal triads resulting in an axis, referred to as liver zonation, along which hepatocyte and sinusoidal function varies. The portal triads consist of mesenchymal cells surrounding the portal vein (PV), hepatic artery (HA), and the cholangiocyte lined intra-hepatic bile ducts (IBD). These are joined to the hepatic canaliculi by the Canals of Hering. The microvasculature of the liver, known as the sinusoid, interfaces the blood supply with the hepatocytes and is also the location of the hepatic stellate cells and Kupffer cells.

The apical surfaces of adjacent hepatocytes form channels known as canaliculi. Bile acids, produced from cholesterol in the hepatocytes, are secreted into the canaliculi (38). The biliary system contains both intra- and extra-hepatic structures both lined with cholangiocytes. The biliary epithelial cells (BECs) of the intra-hepatic bile ducts (IHBD) make up ~3% of the adult liver cell population (39) and drain the canaliculi. Bile flows from the IHBDs of the liver lobes into the hepatic duct, then to the gallbladder in the cystic duct, and finally to the gut through the common bile duct, see Figure 1 (40). Portal vein branches are the sites of IHBD formation, which occurs in several distinct stages. First the hepatoblasts adjacent to the portal mesenchyme differentiate to cholangiocytes, forming rings around the portal vein known as the ductal plate (40–42). The ductal plate then becomes bilayered due to lumen formation at discrete locations between the cholangiocytes and their adjacent hepatoblasts, resulting in asymmetrical ductal structures (43). The lumen surrounding the hepatoblasts differentiate to cholangiocytes, while the remaining ductal plate cells lose their cholangiocyte markers. The ducts mature sequentially along a radial axis as well as on an axis that runs in their direction of growth from the hilum to the periphery of the liver (43, 44).

As well as endoderm derived hepatocytes and cholangiocytes of the parenchyma, the liver contains a mesodermal derived population. The development of the liver requires interactions between the germ layers from the earliest stages and the mesodermal cell types are later incorporated giving rise to the stromal cells of the adult liver, which have crucial functional roles. During human development hepatic specification of the foregut endoderm has occurred by 4 weeks post conception (wpc), with invasion of the adjacent STM at 5 wpc (45, 46). This event mixes the endoderm and mesoderm lineage cells of the liver, with the STM trapped between the growing cords of hepatoblasts (46). The cells of STM will become the mesenchymal cells of the adult liver, the vasculature of the STM gives rise to the sinusoids of the liver, which then further develop by angiogenesis (45, 47, 48). The stromal cells have been shown to interact with other hepatic cell types, for example hepatic stellate cells (HSCs) express HGF (49), interacting with β-catenin, promoting hepatocyte proliferation. The STM derived liver mesenchyme further differentiates forming both transient and adult liver cell types; the mesothelial cells (MCs), sub-MCs, HSCs and myofibroblasts (50). HSCs are found between the liver sinusoidal epithelial cells (LSECs) and the hepatocytes in the space of Disse (51). HSCs are also characterized by their ability to store vitamin A and long dendritic processes that extend along the sinusoid (52).

Blood enters the liver from both the portal vein and hepatic artery, and flows out through the central vein (53, 54), connecting the two are the hepatic sinusoids a microvasculature system specific to the liver. Vasculogenesis of endothelial cell (EC) precursors within the STM leads to the formation of capillaries that later form the LSECs (47, 48). The LSECs line the sinusoid and interface the circulation with hepatocytes. They are highly endocytotic (55) and involved in both development (56) and regeneration (57) of the liver. In the adult liver LSECs make up 2.5% of the parenchyma, surrounding the single cell cords of hepatocytes on their basolateral surface and found in close association with the HSCs (39). After acquisition of a fenestrated phenotype LSECs remain distinct from other endothelial cell types found within the liver, with features such as a minimal basement membrane, loose cell junctions, close association with Kupffer cells and open fenestrations arranged in sieve plates (54, 58). These features allow the transfer of large molecules between the hepatocytes and the bloodstream such as hormones and albumin (39). Functional heterogeneity is observed within the liver parenchyma due to zonal expression of hepatocyte specific genes relative to their position on a portal-central axis, this zonation is also seen in the endothelial cell population (59). An overview of liver architecture is presented in Figure 1.

### The Need for Hepatocytes/Liver Cells?

The utility of liver cell types is hampered by the overarching shortage of primary material, as high quality liver tissue is ear-marked for transplantation purposes where possible, leaving less optimal samples for research use. This is compounded by recent advances, which have increased the pool of viable tissue for transplantation (60, 61). Further difficulties in using, or even benchmarking to, primary cells include the rapid loss of function of hepatocytes and other hepatic cell types during *in vitro* culture, particularly in monolayer (58, 62), as well as limited proliferation potential of HSCs, combined with loss of their quiescent state *in vitro* (63, 64). This is in addition to the much greater loss that is seen when comparing isolated cells to whole *in vivo* tissue activity (65) which makes a broader point about the effectiveness of models composed of dismantled individual parts of a whole system. However, this loss of *in vivo* function and activity, combined with shortage of material, means that even a simple monolayer, single cell type model that can recapitulate features of *in vivo* function would be greatly in demand to replace current systems based on cancer cell lines with limited function such as HepG2 and HepaRG (66). This is also the case for HSC cell lines, which have an activated phenotype and also limited function (67, 68). All of which drives the need for more representative tissue models. To that end there has been a “tour de force” by the stem cell field to produce surrogates of the above using human pluripotent stem cell (hPSC) approaches, these will be expanded upon below.

### The Rational of Differentiation Protocols

iPSC derived organoid models tend to build on protocols first developed for monolayer differentiation with the additional use of extracellular matrices (ECMs), and/or suspension culture to achieve 3D growth. A rational for differentiating PSCs to specific cell type is to mimic the developmental pathway of the desired cell type. Based on developmental studies, the formation of the required cell type can be broken down into discreet stages (though a continuous process) characterized by specific expression patterns. Using this information we can mimic these stages in a spatial-temporal manner *in vitro*, by the addition of growth factors, which has proven an effective process as evidenced by the stepwise differentiation of pancreatic cells through an endoderm stage (69, 70). This rational applied to monolayer hepatocyte differentiation from hPSCs results in shared features between many different groups' protocols (71–75). These methods can result in relatively pure populations of hepatocytes but with the caveat that they more closely match a fetal rather than adult phenotype (6). However, in the context of scaling the high cost of recombinant growth factors makes these approaches prohibitive. To address this, a number of groups have explored small molecules to act as growth factor surrogates. For example in the hepatocyte field a number of protocols have now been published that are as equally effective as their growth factor counterparts (76–78), although still less widely used. The power of producing hepatocyte like cells (HLCs) from patient derived hiPSCs, allows one to model or phenotypically reproduce genetic disorders, in the liver field a number of diseases have been investigated such as glycogen storage disease, alpha-1-antitrypsin deficiency, and familial hypercholesterolemia to name a few (79–81). For an overview of human iPSC disease models see Siller et al. (82). In addition with the emergence of genome editing and gene therapy approaches, there is the potential to correct these disorders (83, 84) both *in vitro* and *in vivo*.

Moving to a 3D culture format offers potential advantages. *In vivo* the polarity of hepatocytes and their arrangement with ECs and BECs in a 3D architecture are necessary for both the endocrine and exocrine functions of the liver (39). To fully recapitulate liver physiology and function using hPSCs may require a mixed population of hepatic cells, which exhibit the correct polarity and arrangement in a 3D context. Additionally, 3D culture techniques have been shown to support primary hepatocytes in a differentiated state (85) for extended periods as compared to monolayer culture (62) and may offer the same advantages to hPSC models.

### Organoids—A Definition and Examples

**Figure 2:**
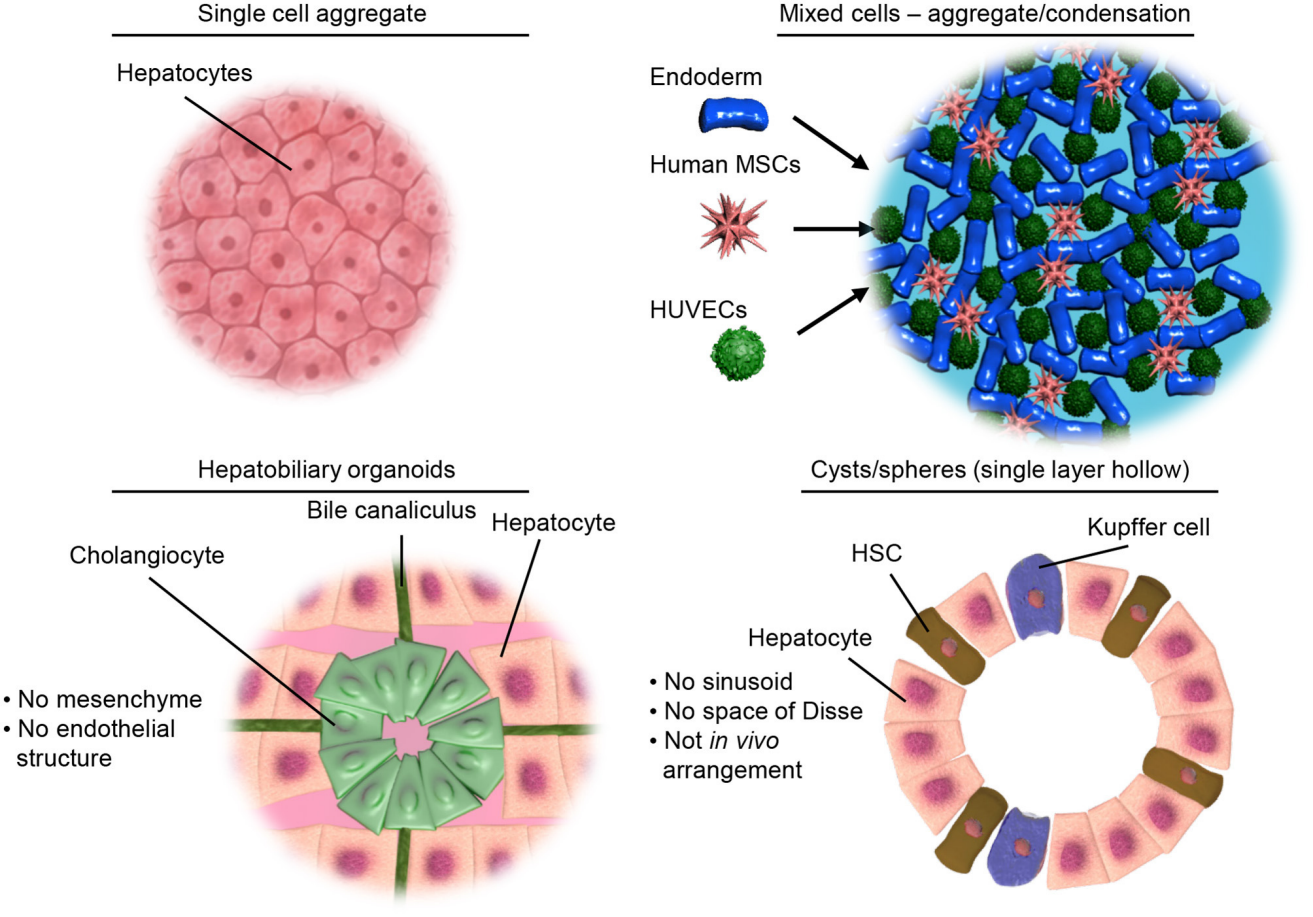
Illustrative examples of different types of 3D culture models. Top left, showing a simple aggregate of a single cell type. Top right, showing aggregates of mixed cell types but limited structural organization as achieved by condensation of pre-differentiated cells. Bottom left, representing the hepatobiliary organoids recapitulating the structure and interactions of the liver parenchymal cells. Bottom right, showing organoids containing both parenchymal and non-parenchymal cells of the liver necessary for modeling inflammation and liver disease.

In the literature there are a variety of 3D culture systems loosely termed “organoids,” some are distinctly organotypic; by virtue of complexity, organization, formation through developmentally recognizable stages (86) or presenting distinct features of an organ (87), while others are formed from aggregates of either single or multiple terminally differentiated cell types, and some lie somewhere in between the two. Here we would like to offer a definition of an “organoid,” as something that undergoes a developmentally relevant process, changing with time, in an autonomous manner resulting in organized heterogeneity and hence complexity, or more succinctly, self-organized 3D cultures derived from stem cells. As an organ is a distinctly organized unit, a parenchyma of cells with associated extracellular matrix, stroma and a specific architecture all of which make functional contributions, they are qualitatively different from homogenous masses of cells or even teratomas as they contain multiple cell types representative of a mixture of tissues. Below we will discuss the different types of 3D cell culture systems available in order of increasing organotypic complexity and we provide an overview of the organoid models discussed below in Table 1 and Figure 2.

**Table 1:** Overview of 3D Liver models.

**References**	**Cell sources**	**Cell types represented**	**Characteristics/ Functionality**	**Applications/Model use**	**Advantages**	**Limitations**	**Developmental stage**
Coll et al. (88)	iPSC HepaRG	•Stellate cell •Hepatocyte	•Retinol Storage •Activatable HSCs •ECM production •Drug metabolism	•Fibrosis response •Toxicity	•Improved phenotype of both cell types over mono culture	•Use of cell line as parenchyma •No tissue-like organization	•Lipid storage and quiescent phenotype suggests mature HSC function •Omics reveals differences to primary cells
Takebe et al. (17)	iPSC	•Hepatocyte •Endothelial cell •Mesenchyme	•Broad hepatocyte function (e.g., metabolism, protein secretion) •Functional vasculature	•Source of potentially transplantable organs, survival improvement after liver failure	•Vascular network •Multiple cell types •Scalable production	•Function relies on *in vivo* transplantation •Lack of biliary or kupffer cell types	•Formed at an early hepatic endoderm stage, followed by subsequent *in vivo* functional maturation
Wu et al. (89)	iPSC	•Hepatocyte •Cholangiocyte •Endothelial cell	•Organized BEC and bile acid production •Hepatocyte functions	•Hepatobiliary functions	•Multiple cell types capable of structure formation and coordinated function	•Low levels of hepatocyte function	•Fetal liver-like organization and functional level
Guan et al. (90)	iPSC	•Hepatocyte •Cholangiocyte •Mesenchymal cell (low abundance)	•Secondary organoid formation •Broad hepatocyte function (bile acid and albumin secretion, CYP metabolism, etc.)	•Liver development and regeneration •Biliary disease-ALGS	•Expandable by secondary organoid formation due to progenitor population •Long term maintenance	•Some HSC activation in routine culture	•Similar to PHH and liver tissue by transcriptome analysis
Ouchi et at. (91)	iPSC	•Hepatocyte •Stellate cell •Cholangiocyte •Kupffer cell	•CYP3A4 expression •Vitamin A storage •LPS response •Hepatocyte lipid accumulation •HSC activation	•NAFLD •Fibrosis response •Wolmans disease	•Multiple cell types •Capable of inflammatory response	•Low Kupffer cell number •Some HSC activation in routine culture •High inter-batch variability	•Fetal-like hepatocyte activity
Huch et al. (92)	Biliary Epithelial	•Hepatocyte •Cholangiocyte	•High CYP3A4 activity •Phase I and II metabolic activity	•A1AT deficiency •ALGS	•Genetically stable •Long term maintenance	•Only parenchymal cell model	•Mixed fetal and adult hepatocyte functions

### Aggregates

It is well-established that in 2D, primary hepatocytes rapidly lose metabolic function (93) and that forming aggregates of these cells has beneficial effects with regards to both longevity and function (94). This has been translated to hPSC derived HLCs by a number of groups including Ogawa and colleagues who formed aggregates and cultured these with the small molecule 8 bromo- cAMP which promoted a more mature phenotype (95). An interesting report from the Suzuki lab (96) utilizing iHEPs generated through transdifferention of mouse dermal fibroblasts exhibited a more mature phenotype on aggregation. This was driven by activation of the HIPPO pathway, leading to the expression of a battery of liver-enriched transcription factors. These models are extremely simplistic and attempts have been made to invoke a more liver like complement of cell types, which can then be used to model states that are dependent on multiple cell types as in liver disease. Liver fibrosis can lead to cirrhosis and hepatocellular carcinoma, as a result such models are of great clinical interest. Coll and colleagues (88) produced iPSC derived hepatic stellate cells (iHSC) using retinol to drive a HSC phenotype from a mesothelial precursor population. When compared to primary material they shared many features such as retinol storage in lipid droplets, activation in response to cytokines and wound healing capability. The iHSCs were then aggregated into 3D spheroids with the immortal hepatoma cell line, HepaRG, which was originally isolated from a female patient diagnosed with hepatocarcinoma and hepatitis C (97, 98). This resulted in iHSCs with a quiescent phenotype along with an improved hepatocyte gene expression profile with respect to the HepaRG cells. Treatment of these spheroids with fibrogenic and hepatotoxic compounds resulted in signs of fibrosis such as iHSC activation, extracellular matrix (ECM) secretion and deposition, highlighting the interaction between the two cell types within the organoids and illustrating their use as a disease and toxicity model. These cultures could potentially be used to investigate mechanism and treatment, as well as investigating the interplay between hepatocytes and HSCs.

The inclusion of two liver cell types demonstrates the advantages of increased complexity in the model over a single cell system. This is exemplified by uncovering a previously unknown hepatotoxic effect of APAP, transmitted via the HepaRG population to activate the HSCs. However, there are clear limitations to the model, most notably a lack of organotypic features, as compared to *in vivo* i.e., the lack of the space of Disse where HSCs would reside, along with a non-physiological ratio of HSC to hepatocytes (HSC making up 50% of the spheroids, compared to 5–8% observed in the liver) (99). In addition HepaRG are an immortalized line and are not functionally equivalent to primary human hepatocytes (PHH), for example widespread gene expression difference were observed when compared to PHH (100). Another critical component missing from this model are the immune cells of the liver, both resident and circulating, which *in vivo* are a source of cytokines released in response to inflammation playing a key a role in fibrosis. Interestingly the authors note a difference in organization of the spheroids as compared to their primary control, where the HSCs formed a separate core, while in the iPSC derived aggregates the HSCs were mixed throughout. This demonstrates that even similar cell aggregates can result in drastically different organization, and invites the possibility that some of these organotypic limitations could be addressed by using iPSC-hepatocyte in place of the HepaRG.

### Condensation

A different and novel approach developed by the Takebe laboratory used a combination of iPSC derived hepatic endoderm and immature non-parenchymal liver cell analogs [mesenchymal stem cells (MSC) and endothelial cells], to mimic the formation of the liver bud (101). In short to mimic the endoderm derived parenchymal cells of the liver invading the mesoderm as *in vitro*. Subsequent investigations showed bud formation was driven by the contractile ability (condensing) of the MSC population, while ECM stiffness was also important (102). The incorporation of endothelial cells allows the formation of a network throughout the bud, leading to anastomosis when implanted after just 48 h. The process of transplantation appeared to promote maturation, implied by loss of alpha-fetoprotein expression, while maintaining albumin expression. The mature buds exhibited adult liver like features, including the formation of hepatic cord-like structures, tight junctions, along with key functions and human specific drug metabolism. In addition increased survival was observed in a drug induced liver failure model, which the authors note as the first demonstration of a functional organ from hPSCs. This MSC driven bud formation acts as a “proof of concept” and was proposed as a potential supply of donor organs for transplant that are both vascularised and functional but with a reliance on *in vivo* maturation. The authors propose the process is recapitulating organotypic interactions between the liver bud cell types, however qualitatively it appears to be inspired by development rather than mimicking the *in vivo* process, resulting in a unique organization between the cell types, lacking liver features such as the sinusoids. This approach appears to be broadly applicable for the generation of a diverse array of tissue buds including: kidney, pancreas, intestine, heart, lung, and brain (102).

An interesting conclusion from these studies was that the use of more immature organ buds appeared to generate functionally better “tissue” after transplantation than those derived from more mature sources. This potentially highlights a specific niche for immature cell types, therefore taking advantage of a common iPSC differentiation limitation (102). The goal of these studies was to generate organ surrogates, however the reliance on primary MSCs and endothelial cells (HUVECs) was a roadblock for standardization and scaling. To address these limitations, with transplantation in mind, the authors developed an iPSC version of the liver bud. For the MSCs an iPSC-STM differentiation protocol was developed generating cells with markers associated with the STM (ALCAM, WT1), while maintaining the ability to drive condensation. Combined with ECs from a previously published protocols and custom large scale culture plates they successfully generated 10^8^ buds which were subsequently transplanted and shown to give a significant survival improvement in a liver failure model (17).

Whilst this system generates vascularised functional parenchymal tissue, there are some clear organotypic limitations to the liver bud model. Notably, the absence of cholangiocytes and biliary structures would be expected as input endoderm was derived from HE cells. This may be a consequence of starting from a monolayer differentiation of iPSC-HE, efficiently directing all the cells to the same fate and removing the required heterogeneity. The inability to model the biliary system also raises the question of what happens to the bile produced in these liver buds post-transplantation? The absence of a portal area, where the biliary tree is located *in vivo*, may also prevent establishment of zonation in the liver buds. An additional component not described is LSEC specific features or heterogeneity expected within the liver endothelial cells, which may be a consequence of following a non-liver specific iPSC-EC based protocol, or the use of human umbilical vein endothelial cells (17). Whilst an STM like population was used in this follow-up model (17) it was lacking a description of potential stellate specific functions or activity, such as retinol storage or ability to become activated, along with the organization seen between these cells types in the liver, the sinusoid and space of Disse etc. These limitations may preclude the use of this system to model diseases involving inflammation, fibrosis or the biliary system, admittedly not proposed by the authors. These features are however available in other models discussed below revealing the need for a plurality of models for different uses with their various advantages and disadvantages.

### Hepatobiliary Organoids

A perhaps more physiological model is described by Wu and colleagues (89). The authors produce hepatobillary organoids by adapting established hepatocyte differentiation, to induce both endoderm and mesoderm tissues in the same culture, relying on transforming growth factor beta (TGFβ) induction to form separate hepatocyte and cholangiocyte populations from a single source. The hepatobiliary organoids were then matured using a standard base media supplemented with a proprietary cholesterol (MIX). This model recapitulates some of the stages of hepatogenesis, such as formation of bipotent precursors and their subsequent resolution to both lineages (as well as the formation of endothelial cells) and the organization of these cell types into appropriate structures such as lumen surrounding cysts and tubes. The BEC were shown to have an apical basal polarity and cilia, while bile acid was found within cystic structures. This is indicative of a functional multicellular system, as the bile would need to be produced by the hepatocytes flowing through canaliculi like structures before being emptied into and concentrated in the ductal cavity. The biliary cells first formed rings and then further developed in to tubular or cystic structures growing out of the culture. The formation of these structures was shown to be linked to NOTCH signaling and was hypothesized to be associated with the previously noted endothelial cells, reminiscent of the cross talk between cell types observed during development *in vivo*, where the mesenchyme plays this role (103–105). The resulting culture showed increased expression of markers of maturity for both hepatocyte and biliary lineages, however they share the same shortcomings of many iPSC derived progeny, presenting with low levels of CYP450 activity, whilst matching or exceeding those of fetal liver, they were a fraction of adult liver levels.

The above model does fit the organoid label, exhibiting complex multi-cell type features such as bile production, flow, and concentration in tissue approximate structures. The approach itself appears rather unorthodox as the cells were cultured as a mono-layer and subsequently allowed to overgrow during the differentiation procedure to form 3D masses, in contrast to more common methods of embedding within an ECM. The authors highlight the lack of need for a supporting mesenchyme or fibroblast cell types and the absence of ECM or biomaterial, ultimately reducing cost, and complexity. However, the lack of mesenchymal cell types might not be an advantage for making a comprehensive organoid.

Another hepatobiliary model has been developed by Guan et al. (90). They used iPSCs to generate 3D organoids following stages resembling *in vivo* liver development which again contain both hepatocytes and cholangiocytes organized into epithelia and duct-like structures surrounding a lumen, referred to as hepatobiliary organoids. The hepatobiliary organoids were formed using iPSCs in suspension in the presence of an ECM, matrigel, and a cocktail of growth factors and small molecules, allowing the formation of complex organoids, *via* posterior foregut, over a 20 day period, these were termed HO1s. These organoids were then dissociated to single cells and embedded into matrigel leading to the formation of secondary organoids termed HO2s. These organoids were shown to follow developmentally relevant liver stages; endoderm, liver bud, hepatoblast formation. The HO1 stage generated three types of spheroid, parenchymal, ductal, or a mixture of both. Transcriptome analysis of HO1 group revealed a greater degree of similarity to liver tissue than to primary hepatocytes ascribed to the presence of cholangiocytes. Interestingly the HO2s showed signs of improved maturity such as increased expression of *CYP3A4, TTR*, and *TDO2* with decreased expression of *AFP*. Both HO groups exhibited liver functions such as production and accumulation of bile acids, albumin secretion, CYP3A4 metabolism and were effective at secondary organoid formation, a route for potential scalable culture. As in the Wu protocol described above non-parenchymal cell types were observed but minimally characterized, here the authors identified a mesenchymal population comprising 1–2% of the organoids, by virtue of αSMA staining. This marker is associated with HSCs during the development of the liver *in vivo* and it seems a potential oversight to not further characterize these cells and consider their effects within the culture.

The ability to form secondary organoids (HO2s) was proposed as a model for liver growth (as well as regeneration), which occurs postnatally by an increase in lobule number (106). The authors identified a SOX9/CK7+ population in the HO1s, these markers are shared by a progenitor cell type thought to be capable of regenerating the liver *in vivo* (107). These cells could be expanded and passaged multiple times under specific growth conditions. Further characterisation lead to speculation these were either a liver progenitor or reactive cholangiocyte population, based on expression of CK8, HNF4α, CK19, EPCAM, and SOX9, along with decreased hepatocyte marker expression. All of which suggest dedifferentiation to an earlier developmental stage. Interestingly this population could then be differentiated to hepatocytes and cholangiocytes, with a small population remaining positive for the above progenitor marks, associated with the ductal structures. This highlights a similarity to an *in vivo* conjectured liver progenitor population associated with the canals of herring (107), suggesting a recapitulation of this niche occurs as part of the formation of a hepatobiliary organoids.

### Adult Organoids

Liver organoids formed from primary cells have been generated by Clevers and colleagues (87, 92) using EPCAM+ biliary epithelial cells, this expands on previous work using a LGR5+ stem cell population isolated from the intestine (7, 87). They show that these organoids recapitulate some of the epithelial features of the original organ; arranged as polarized cysts, and though derived from the ductal cells the organoids show hepatobiliary features and could be directed to a hepatocyte-like phenotype (92). These organoids were clonally derived, expandable and could be maintained for several months. More recent work in the area has developed hepatocyte derived organoids (Hep-Orgs) from both adult and fetal liver (108), which resulted in a more distinct hepatocyte (as opposed to biliary or hepatobiliary) phenotype. These include structural features such as MRP2^+^ bile canaliculi and tight junctions along with albumin secretion and CYP3A4 activity levels comparable to those from primary human hepatocytes (PHHs). Levels of AFP secretion were initially high but were found to decrease over time which supports the authors hypothesize that the Hep-Orgs are derived in a process analogous to the regenerative response of the liver, as the usual fetal associated AFP secretion is also induced after hepatectomy. The absence of cholangiocyte function was demonstrated by a lack of the MDR1 activity seen in the Hep-orgs while there was only a low number of cholangiocytes generated.

A limitation of the adult cell derived organoids is the lack of heterogeneity in the starting populations, as they are expanded from epithelial cells this removes the chance for endothelial or mesenchymal cell types, which would potentially contribute to future complexity and a more organotypic model. This is in turn has implications for their potential use in modeling diseases such as fibrosis, NAFLD and HCC where inflammation and the cellular microenvironment are involved (109, 110), or development that relies on the presence of multiple cell types and lineages. Their suitability and capability to model monogenic disorders such as ALGS and A1AT deficiency has already been demonstrated (92). Points worth further investigating are whether the primary derived organoids form following a process that more closely resembles regeneration rather than development, with the data from Hep-Orgs suggesting the former, and whether a more tissue-like parenchymal model can be developed that includes both hepatocytes and cholangiocytes organized as *in vivo* (e.g., with duct structures). A clear disadvantage of organoids derived from primary sources is the difficulty in acquiring and harvesting the required material in comparison to using PSCs. While use of iPSC derived organoids share the advantage of being derivable from the desired donor or genotype but in a non-invasive manner. Where the adult derived organoids seem to have a clear advantage is in the genomic stability and potential longevity of their cultures (87, 92). With maintenance of functional cultures demonstrated for up to 1 year is in contrast to the primary material that rapidly lose function, as well as the majority of iPSCs derived organoids which have shorter functional windows. The genomic stability of the adult organoids, seen at both the sequence and chromosome levels, is also in contrast to that of iPSC organoids that often harbor genetic abnormalities thought to be caused by reprogramming (111). The process of reprogramming and the generation of useable patient specific iPSCs is also a long and costly addition when compared to generating primary organoids. More in depth reviews on adult liver organoids and their use in disease modeling can be found here: (112, 113).

### Inclusion of Non-parenchymal Cells

A more organotypic model than the mostly parenchymal hepatobiliary organoids should include significant numbers of the stromal cells of the liver an example of which has been demonstrated by Ouchi et al. (91). Their organoids are produced in a similar manner to that described above, via a foregut differentiation step, but generating mesenchymal as well as epithelial cell types, which then have the potential to become the necessary stromal cells. These foregut spheroids were embedded in matrigel and further differentiated to a hepatic lineage and the resulting termed human liver organoids (HLOs). RNAseq revealed their hepatic nature with respect to lipid homeostasis genes, though they still form a distinct cluster away from both hepatocytes and fetal liver. Both immunostaining and FACs analysis showed populations with parenchymal markers as well as those associated with mesenchyme and macrophages, though the high expression of EPCAM suggested a distinctly early phenotype for the hepatocyte-like cells. Single cell RNAseq (scRNAseq) used to further analyse the HLOs found that the majority of the cells (~60%) were hepatocytes and that they expressing portal over central markers, although these markers may also represent an earlier pre-zonal hepatocyte rather than a specific portal phenotype as hepatocyte zonation continues to develop through adolescence (114). Approximately 30% of the cells identified through scRNAseq had a HSC-like expression signature, with a small number of biliary and even smaller number of Kupffer cells, representing a considerable organ specific cell type diversity. Basic functional activity of the hepatocytes revealed inducible CYP3A4 expression though further characterisation was lacking perhaps due to the fetal characteristics of the hepatocyte-like cells. The HSC cells were shown to be able to store vitamin A after retinol treatment, a crucial function of the stellate *in vivo*. To investigate the activity of the Kupffer population the authors treat their organoids with LPS and see an activation response in the stellate population which is interpreted as being Kupffer mediated, though the same assay produced a similar response in the Coll paper described above which did not contain a Kupffer population suggesting that such a response is not indicative of a macrophage population. Additionally, LPS directly activates HSCs through TLR4/CD14 (115), by enhancing the effects of TGFβ (116) showing its unsuitability as an assay to distinguish between Kupffer and HSC mediated responses.

## Application of Organoids in Disease Modeling and Regeneration

### Biliary Model

An example that highlights the current uses and future potential of liver organoids in disease modeling and regenerative medicine is that developed by Guan et al. described above. Its utility is exemplified by modeling Alagille syndrome (ALGS), a defect caused by impaired NOTCH signaling. The study aimed to elucidate the mechanism of the disease and the cell types involved. Hepatobiliary organoid (HOs) were generated from ALGS patient iPSCs and via CRISPR engineered lines and compared to controls. This revealed an absence of duct structures and reduced cholangiocyte marker expression from patient organoids. Further, reduced expression of Jagged 1 (JAG1) expression was observed during the HO development, along with an absence during the structure forming stage compared to control. JAG1 is a notch ligand and mutations in this gene lead to ALGS and impaired bile duct formation. The ALGS HOs lacked the ability to form secondary organoids, indicating an impaired regenerative capacity. This was further validated by rescue using CRISPR in isogenic controls. Demonstrating the principle that genome engineering can be used to generate and repair disease lines. This allowed unique insights into the mechanistics of ALGS revealing that it is not caused by immune mediated damage and explaining the timing and pattern of bile duct paucity *in vivo* as the inability of the ducts to spread into the newly forming lobes of the liver. In contrast to their initial fetal formation, which is driven by mesenchymal tissue. Leading the authors to conclude that postnatal bile duct expansion is driven by the hepatoblasts.

### Steatosis Model/Fibrosis

Over the last decades the incidence of non-alcoholic fatty liver disease (NAFLD) has risen sharply worldwide. Currently, a number of model systems are utilized including animal based models [reviewed in Lau et al. (117)] and primary or immortalized cell-based models [reviewed Müller and Sturla (118)]. The above however have limitations in that the physiology of animals is significantly different to that of humans, combined with the ethical considerations and in the case of primary cells very limited in there availability. The iPSC field has been evolving with a number of models being developed to investigate liver steatosis and liver diseases, however the majority of these exploit standard 2D culture systems to investigate NAFLD (119, 120). This has led to the field to develop more organotypic models that reflect the organ. Examples include the development of a hybrid iPSC and immortalized cell aggregate system by Coll et al. (88) and the more sophisticated 3D organoid model developed by Ouchi et al. (91) both described above.

The Ouchi et al. hepatic organoid model shows the advantages of HSCs in an organotypic setting that in combination with Kupffer cells, the resident liver macrophage, highlights the modeling complexities attainable in these systems. The authors developed multicellular organoids with hepatocyte, stellate, and Kupffer analogs, which in response to free fatty acid treatment show signs of steatohepatitis, including steatosis, inflammation, and fibrosis. They also demonstrated that organoid stiffness correlates with fibrosis severity and recapitulated steatohepatitis using iPSCs derived from patients with a specific genetic dysfunction and subsequently rescued it showing the potential for their organoids to be used to model and investigate diseases. Treatment with free fatty acids was found to increase lipid accumulation by the hepatocytes of the HLOs, with oleic acid treatment causing hepatocyte enlargement (ballooning), a hallmark of steatohepatitis. This treatment caused increased expression of IL6, IL8, and TNFα in the HLOs. After 5–7 days of continuous steatohepatitis induction, the HLOs showed markers of fibrosis such as increased αSMA staining and collagen deposition along with decreased vitamin A storage. Interestingly this steatosis response to oleic acid was found to be absent from aggregate spheroids generated from cell lines suggesting it is a feature specific to an organotypic system. Using patient derived iPSCs the authors modeled Wolmans disease, which is a genetic disorder that causes lipid accumulation followed by steatohepatitis and fibrosis, in the HLOs. Currently only treatable using an expensive drug, the authors were able to reverse this phenotype by treatment with FGF19 supressing lipid accumulation and increasing the survival of the steatosis HLOs. Thus, demonstrating the utility of their steatosis and fibrosis model and highlighting a potential alternative treatment for the disease.

The incorporation of stellate and Kupffer cells into the model gives it a unique niche as well as the demonstration that some features are unique to organotypic models. The authors also highlight that mixed co-culture aggregates such as the Coll model and the Takebe liver bud model show signs of inflammation and fibrosis during routine culture which they speculate is due to unsuitable culture conditions for the multiple cell types, which will limit their use in inflammatory modeling. The array of cell types in the HLOs gives lots of potential for future use and development lacking only an endothelial population from previously demonstrated models. Though other hepatic functions besides lipid homeostasis were not well-characterized and the organization of the cell types is left unreported, such as do biliary cells form structure, how and where are the Kupffer cell located etc? Also noted by the authors is batch variability, particularly with respect to αSMA positive cells, as a potential limitation of the model.

### Liver-On-Chip

The use of chip based or microphysiological (MPS) culture systems offers many promising advantages in terms of control of environment, reproducibility, feedback and automation when compared to traditional manual cell culture. Many variants of *in vitro* liver model have and can be translated to these continuously advancing culture systems, though currently most examples use aggregates of primary cells or hepatic cell lines (121–123) which represent a more simplistic cellular model than the organotypic complexity approached in the above described organoids. There are also many description of liver-chips which use the physical structure of the chip itself, or bioprinting, to force organization on multiple different cell types (124–126) thus engineering organotypic complexity into the system, these however fall outside the organoid topic of this review.

### Overview of Optical Microscopy Imaging of Organoids

An area developing in parallel with advancement of organoid technology is the rise of novel volume high-resolution optical imaging approaches (127–132). Powerful imaging approaches will allow the probing of cellular and subcellular complexity within organoids. Furthermore, volume imaging methods will potentially enable characterization of the architecture of whole-mount tissues in 3D (132). The superior qualities of 3D imaging are of a great importance to further our understanding of cellular composition, cell shape, cell-fate decisions and cell–cell interactions of intact biological samples, especially pertinent for the organoid field (127, 131, 132).

### 3D Imaging

It is worth noting, that organoids are 3D objects and may be relatively large in size. Thus, it is demanding to perform high volume 3D imaging of organoids while minimizing phototoxic effects and supporting their physiological integrity as experimental models. On one hand, there is a need to visualize an organoid as a whole object and, on the other hand, probe the cellular complexity of organoids at the subcellular level. Imaging of relatively large objects requires deep 3D tissue imaging. Ideally, one desires to achieve a micrometric optical sectioning capacity and sub-micrometric spatial resolution in combination with a large field of view combined with a high frame rate, and low level phototoxicity (133). However, all techniques have their advantages and limitations. In this section we briefly discuss existing technologies in organoid imaging along with their limitations.

In order to achieve imaging of large and sensitive specimens, like organoids, it is essential to minimize exposure of the sample to light and perform fast and contrasted signal recording. Indeed, organoids can be up to 3 mm in size (134). A major problem in optical imaging of thick 3D biological samples is the loss of contrast due to multiple scattering (135). Thus, wide-field epifluorescence microscopy is unsuitable for such purposes having a maximum 700 μm depth of penetration (136, 137). *Confocal laser scanning microscopy (CLSM)* systems improve the depth of imaging (up to 1,000 μm), but they are relatively slow in making image (~1 s for a single frame) (127, 138). Indeed, for initial imaging of fixed samples a basic confocal microscope is sufficient (139). One can derive basic information about organoid structure and function using CLSM systems. However, major drawbacks of CLSM systems are out-of-focus fluorescence excitation, out-of-focus photo-bleaching and photo-damage (133).

Implementation of a spinning disk scanner containing a set of confocal pinholes facilitates high-speed acquisition and removes the speed-limiting disadvantage of a single-beam CLSM (140). As a result, *spinning disk confocal microscopy (SDCM)* significantly reduces phototoxicity, and additionally enables high-resolution imaging by utilization of low-noise combined with high-dynamic-range detectors [e.g., charge-coupled device (CCD) cameras] (141, 142). Spinning disk confocal systems in combination with silicone oil-immersion objectives allow super-fast image acquisition (up to ~0.5 ms for a single frame) (143). Moreover, silicone oil-immersion objectives, due to significant reduction of spherical aberration, produce brighter and higher resolution 3D images of biological samples, especially at deeper sample depths (improving depth of imaging up to 2,000 μm) (143). This technique has enabled high-contrast 3D live-cell imaging of a mouse embryo over ~4 days for example (143).

*Multiphoton microscope systems* outperform CLSM having much deeper levels of imaging to about 2,000 μm below the surface (144, 145). Multiphoton systems utilize much longer wavelengths of light to excite fluorescence specimens and scatter less as they scan through tissue (144, 145). Interestingly, if one combines multiphoton microscope with ultra-long-working-distance optics and proprietary clearing reagents, it is possible to image up to 8,000 μm (8 mm) deep in fixed specimens (146).

As it stands for now, *light sheet fluorescence microscopy (LSFM)* is the best technique to rapidly acquire optical sections in thick specimens (147). LSFM utilizes a laser light-sheet that illuminates a plane of optical section and views tissues with subcellular resolution (148). Due to such fast optical sectioning LSFM is able to image thicker tissues (>1 cm) with reduced photobleaching and phototoxicity of a specimen (148–150). However, spot-scanning microscopes still outperform LSFM in the axial resolution (133). Additionally, to get light-sheet image sampling one needs an orthogonal arrangement of excitation and collection objectives (133). This creates another limitation of LSFM, to achieve proper image quality it is important to have non-standard procedures for embedding and holding the sample (133). This in turn creates challenges in experimental variability, especially with samples submerged in liquid media.

## Discussion

Whilst many 3D liver models are described as organoids these are clearly not all equally organotypic, a point previous reviews have not addressed. Our aim here is to give an overview of these different models whilst distinguishing and clarifying these qualitative differences between them and to provide a framework to assess and compare future studies. We would also hope to highlight and persuade the reader for the need of organ level complexity, as we believe it to be crucial to investigate features not possible in more simplistic systems.

The different types of models explored above reveal that more physiological approaches are important to accurately recapitulate the complexities of disease/development, which involve multiple cell types and their coordination into physiologically relevant structures. By comparing these different studies we see that the complexity demonstrated by multiple cell systems allow improved toxicity and metabolism studies, as well as engraftment, fibrosis, and inflammation models. Additionally the mimicking of liver structure and organization can be used to investigate development and physiology and hence disease that arise through disruption of these processes, here made particular use of by the hepatobiliary models (Alagille syndrome). These examples serve to highlight the inadequacy of 2D liver models except for use in specific circumstances and whilst more simple models can often fulfill the purpose they are designed for, complex structure and physiology mimicking systems present many possibilities for repurposing and future applications.

Drug metabolism by CYP450 enzymes is used in several of these examples as a way of demonstrating hepatic phenotype/maturity and hence successful differentiation of this cell type. However organoid models are capable of more sophisticated uses which may aid drug development, however the field is currently hampered *in vitro* by the use of low activity hepatocytes [e.g., Ouchi et al. (91)].

An interesting insight raised by the Takebe laboratory is signs of inflammation in their organoid models, a feature we also have observed (151), resulting in inter-batch variability with respect to signs of HSC-like cell activation. This could have effects on the use of the models for fibrosis, inflammation, toxicity and disease studies all of which could be envisaged to involve cytokine response. Suggesting the need to develop culture conditions that can provide a quiescent state *in vitro*.

A more general question still to be addressed when examining organoid models is whether the *in vitro* organoid forming processes are following *in vivo* development or a distinctly different culture system induced “development.” If these processes are found to vary between different protocols then each will have to be assessed independently as to whether the model being used complements and enhances *in vivo* studies.

One of the most prominent and perhaps most challenging limitations of current organoid models and PSC differentiation *per se* is the tendency for the expression of immature or fetal markers, for example the expression of AFP or high levels of CYP450 3A7, in differentiated hepatocytes and an overall lack of liver zonation. There are many potential and overlapping causes for this such as; too short culture times–as zonation and liver development continue in the postnatal period and through adolescence taking years to become established *in vivo*, lack of required complexity in the model and also a lack of exposure to a microbiome, although limited attempts to recreate microbial cues have been attempted with lithocholic acid and vitamin K_2_ albeit with a focus on maturity and not zonation (152). These possible causes are not limited to PSC derived liver models as isolated hepatocytes are known to rapidly dedifferentiate *in vitro*, a problem which may share a common cause. It seems reasonable to speculate therefore that as organoid models increase in complexity, becoming more tissue-like and more closely mimicking the physiological environment, this may aid in their maturation too. However, a developmentally immature phenotype could be a feature inherent to using PSCs due to residual expression of PSC gene regulatory networks in differentiated cells, as determined by comparison of pre-existing datasets covering the differentiation of different cell types from ESCs (153). Overcoming this limitation is paramount for *in vitro* use and could be transformative for the use of PSC derived liver organoids in metabolism and toxicity studies as adult hepatocytes currently out perform PSC derived cells by several orders of magnitude. The recent study from Boon et al. (154) shows that at least a part of this maturation conundrum, in a liver specific setting, can be addressed by altering the metabolic status of the cells by the addition of extracellular amino acids at levels beyond nutritional needs. This pushes both iPSC derived hepatocytes and other hepatic cell lines to a phenotype closer to that of primary human hepatocytes, of particular interest in terms of xenobiotic metabolism and toxin sensitivity.

The scarcity of primary tissue means *in vitro* organoid models are often lacking in functional comparisons to primary hepatocytes or tissue. This is the case for many organoid studies were functional attributes are limited, often compared to immortalized lined such as HepG2 or to cryopreserved hepatocytes (89, 92). Some studies have no functional data comparison to primary material (91), and many studies only look at the usual suspects albumin and CYP3A4 (90). Also functional niches are highlighted were adult levels are achieved in the case of albumin and urea levels (17), or albumin and CYP3A4 (108). Additionally, for example Albumin secretion levels are often used as a proxy for differentiation quality but are rarely the actual function desired in the model. CYP3A4 function is often noted (see above) however other peri-central associated markers in contrast are lacking in many characterisations, which along with phase II enzymes and non-CYP based metabolic enzymes would be required to model xenobiotic function, this is potentially as a lack of zonation may preclude their high level expression. These functional assays are particularly relevant to the liver as toxicity and xenobiotic metabolism are key to drug development, which is often the suggested goal of liver organoids. Functional limitations and broad equivalence between iPSC hepatocyte differentiations has long been noted (73), however the improvements provided by some of the organoid models here (multiple cell types, *in vivo*-like organization, long term culture maintenance) may provide the advances required for greater functional maturation, and though this has yet to be comprehensively demonstrated there are signs that this is the case [e.g., transcriptomic comparisons (90, 91)]. As the current gold standard is PHH activity rather than whole liver functionality and cell death or damage is a common readout, any such comparison with organoids for toxicity may benefit from a hepatocyte specific marker to allow an accurate comparison, such assays have already been investigated with co-cultures in mind (155). This lack of parity with respect to characterisation highlights the necessity for standardization in the context of basic functional attributes of organoids that will be accepted and applied by the research community.

Another limiting factor for future use is the scale of production and its associated costs. Most differentiation protocols are not optimized for industrial scale production/use and heavily rely on expensive recombinant proteins, which could be prohibitive. This is also a consideration for potential clinical applications, where the standard therapeutic scale is considered to be 10∧9 hepatocytes or more per patient (156). Undefined conditions including serum use, growth factors and ECM present as barriers to transplantation and regenerative medicine. A future idealized organoid model would therefore be generated via small molecules and both serum and ECM free.

Other ingredients yet to be incorporated include red and white blood cells, save for the Kupffer cells, these cell types may be expected to help form part of the complexity required to mimic *in vivo* function and additionally follow correct development of a liver model, as the liver is an early site of haematopoiesis during its development, along with the vasculature of the liver. Whilst endothelial cells have been noticed, their *in vivo* diversity and complexity have yet to be captured wherein they are organized into veins, arteries and zonated sinusoids. Achieving this may be critical in establishing hepatocyte zonation and mimicking the acinar unit.

A consideration of non-liver organoids can reveal imminently achievable possibilities directing future work as well as the potential of combinatorial organoid models leaving room for rapid advancement into areas previously covered. An example of this is the innervated intestine organoids from Workman and colleagues (9). The enteric nervous system (ENS) is a crucial functional part of the intestine and was incorporated into human intestinal organoids by inclusion of neural crest cells resulting in an innervated organoid with ENS functions capable of modeling genetic diseases. A similar approach may result in improved liver organoids, as the human liver unlike that of rodents each hepatocyte is in contact with a nerve cell making innervation a crucial part of the *in vivo* liver architecture so far neglected (157). In fact innervation has been shown to be a driver of maturation in a number of tissues including muscle and pancreatic islets (158, 159). Therefore, innervation may well be one more piece in the puzzle of maturation.

A recent development is the generation of boundary organoids, Koike and colleagues describe hepato-biliary-pancreatic organoids (falling slightly outside of the scope of the main body of this review) formed by combining individual foregut-midgut organoids, then allowing them to differentiate together, essentially recapitulating an area of the embryo as opposed to a single organ (160). This adds extra dimension of complexity to the model but invariably removes specificity. With this comes the caveat that scaled up production of this technique seems more complicated and the resulting organoids currently showed limited maturation and function. They are perhaps best suited, as suggested by the authors, to studying development and its associated diseases. Combining distinct organoid tissue models in co-culture is another area of potential interest particularly with respect to toxicity and drug development. Here the liver organoids could be paired with any other organ, by integrating the xenobiotic biotransformation potential of the liver allows metabolite influence to be examined on the target tissue directly. This is perhaps best realized in the field of “Organ on a chip” (OoC), or microphysiological devices. Currently the majority of systems use simple cell models, therefore the integration of organoids would potentially benefit both the complexity and the utility of the chip. OoC devices would allow closer control of the cell environment, further enhanced by integration of sensors/online readouts allowing continuous measuring and sampling using techniques such as mass spec and electrophoresis (161). Microfluidics can be used to more precisely mimic *in vivo* conditions in the context of oxygen, flow rate and sheer stress, important parts of the hepatic niche. With the clear potential to investigate a portal-central vein axis, along with the establishment of zonation, gradients of signaling and oxygen. Such devices will allow combinations of different organoids in pairs, or more, to examine interactions with drugs by combining liver with the target organ(s) e.g., brain to study effects of drugs on neurons or nervous control of hepatocyte function, pancreas to examine energy and endocrine systems, or gut to investigate the entero-hepatic axis. These co-cultivation experiments require the presence of the necessary machinery for the organ-crosstalk and therefore add another layer of organoid characterization. For example, exploring signaling between adipose tissue and liver organoids would require the correct repertoire of signaling receptors and secretion profiles (162). These technologies can be further refined toward personalized medicine, using patient specific iPSCs to derive the organoids and combining multiple such organoids on a chip resulting in the ultimate *in vitro* model for drug testing and disease modeling.

The application of organoid technology to study disease, development etc., requires functional readout in different formats including biochemical, genomic and transcriptional. As discussed above visual assessment has been co-developing with 3D culture systems and has come on leaps and bounds. Recent advancements in fluorescence microscopy clearly state that the diffraction barrier is no longer the ultimate challenge in obtaining biological super-resolution images (163). For example, MINFLUX nanoscopy was reported to achieve unprecedented resolution in the range of 1 to 3 nm for structures in both fixed and living cells (163). However, the challenge for high-resolution, super-resolution and even standard confocal optical microscopy is photo-damage (127, 164, 165). Unfortunately, to date live-cell super-resolution localization microscopy has largely ignored phototoxic effects (166). In fact, articles mentioning terms related to fluorescence microscopy revealed that <6% of articles mention phototoxicity (166). To achieve super-resolution requires high irradiation intensities (kW cm^−2^ range) (165, 167). Such high illumination intensities generate toxic free radicals from exogenous dyes and endogenous chromophores, leading to cellular damage and cell death (165, 168–170). More specifically laser-damaged mitochondria are a source of oxidative stress triggering cell death (165, 168, 169). Therefore, a fundamental understanding of the mechanisms of light-induced phototoxicity will provide the approaches to minimize photo-damage. The field is already addressing some of these issues such as using reactive oxygen species (ROS) scavenging buffers (168, 169, 171) and also minimizing pre-imaging stress of cells, by limiting overexpression of tag proteins and titration of fluorescent dyes (165, 172). Indeed even simple adjustments such as limiting laser intensities (173), performing microscopy under optimal cell culture conditions (165, 172) and the use of far-red excitation wavelengths can all reduce photo-damage (168), especially when then combined with increased scanning speed (138, 172, 174). These technological limitations will continue to co-develop with organoid research being addressed as and when required.

## Author Contributions

GS, SH, OL, AD, SB, and RV wrote the manuscript. GS and SH edited and approved its final version. All authors contributed to the article and approved the submitted version.

## Conflict of Interest

The authors declare that the research was conducted in the absence of any commercial or financial relationships that could be construed as a potential conflict of interest.
